# Pre-operative Assessment of Shoulder Pathologies on MRI by a Radiologist and an Orthopaedic Surgeon

**DOI:** 10.5704/MOJ.2403.006

**Published:** 2024-03

**Authors:** S Winkler, B Herbst, K Kafchitsas, P Wohlmuth, P Hoffstetter, MJ Rueth

**Affiliations:** 1 Department of Orthopaedic Surgery, Asklepios Hospital Lindenlohe, Schwandorf, Germany; 2 Department of Research, ASKLEPIOS Proresearch, Hamburg, Germany; 3 Department of Radiology, University of Regensburg, Regensburg, Germany; 4 Department Sports Clinic, Sportklinik Fichtelgebirge, Markredwitz, Germany

**Keywords:** MRI, biceps tendon, surgeon, shoulder, radiologist

## Abstract

**Introduction:**

Pathologies of the shoulder, i.e. rotator cuff tears and labral injuries are very common. Most patients receive MRI examination prior to surgery. A correct assessment of pathologies is significant for a detailed patient education and planning of surgery.

**Materials and methods:**

Sixty-nine patients were identified, who underwent both, a standardised shoulder MRI and following arthroscopic shoulder surgery in our hospital. For this retrospective comparative study, the MRIs were pseudonymised and evaluated separately by an orthopaedic surgeon and a radiologist. A third rater evaluated images and reports of shoulder surgery, which served as positive control. Results of all raters were then compared. The aim was an analysis of agreement rates of diagnostic accuracy of preoperative MRI by a radiologist and an orthopaedic surgeon.

**Results:**

The overall agreement with positive control of detecting transmural cuff tears was high (84% and 89%) and lower for partial tears (70-80%). Subscapularis tears were assessed with moderate rates of agreement (60 – 70%) compared to intra-operative findings. Labral pathologies were detected mostly correctly. SLAP lesions and pulley lesions of the LHB were identified with only moderate agreement (66.4% and 57.2%) and had a high inter-rater disagreement.

**Conclusion:**

This study demonstrated that tears of the rotator cuff (supraspinatus, infraspinatus) and labral pathologies can be assessed in non-contrast pre-operative shoulder MRI images with a high accuracy. This allows a detailed planning of surgery and aftercare. Pathologies of the subscapularis tendon, SLAP lesions and biceps instabilities are more challenging to detect correctly. There were only small differences between a radiologic and orthopaedic interpretation of the images.

## Introduction

Shoulder pathologies are very common, especially in athletes with a high overhead activity (i.e. ice hockey, tennis, baseball)^1-3^. Asymptomatic professional baseball players had a prevalence of 79% of labral abnormalities on MRI images^4^. Full thickness tears of the rotator cuff have a prevalence in the general population between 11.75%^5^ and 22.2%^6^. It is even higher in overhead athletes due to the elevated torques and forces^7^. Partial tears of the rotator cuff have an overall incidence of 18-20%^5^ which seems to increase with age with reports of 4% under 40 years and 26% over 60 years^8,9^. It is the authors’ clinical experience that many patients already had a current, non-contrast, MRI examination of the affected shoulder when consulting a specialised orthopaedic surgeon. It is crucial for the surgeon to interpret MRI findings correctly to better educate patients and plan possible surgery and aftercare. In the authors’ view the radiologist’s report demonstrates, not infrequently, a slightly different evaluation. A possible explanation could be the missing physical examination of the patient and limited opportunity of reassurance by intra-operative findings by the radiologist.

We designed this retrospective analysis of our shoulder surgery cases to explore the accuracy of pre-operative assessment of MRI findings and to detect whether there are differences between a radiologist and an orthopaedic surgeon.

## Materials and Methods

More than 200 shoulder arthroscopies are performed each year in our specialised department. We retrospectively screened for patients who had both a standardised non-contrast shoulder MRI in our hospital and afterwards a shoulder arthroscopy in our department between 2016 and 2017.

Sixty-nine patients (n=69) were identified and included. This ensured uniform shoulder MRI images and surgical examination of all participants. All study patients underwent a non-contrast enhanced MRI examination of the shoulder joint with a standardised examination protocol. A 1.5 Tesla MRI Unit [Siemens Healthcare, Erlangen; Germany] was used with a dedicated anatomical shoulder coil.

Coronal: fat saturated protone weighted turbo spin echo (PD FS) TR 2700; TE 43; matrix 320x320; acquisition time 2:55; FOV 100x100mm, slice thickness 3mm.

Coronal: T1- weigthed spin echo (T1 SE) TR 577; TE 13; matrix 320x320, aquisition time 3:05; FOV100x100, slice thickness 3mm.

Axial: fat saturated protone weighted turbo spin echo (PD FS) TR 3640; TE 50; matrix 320x320, acquisition time 3:30; Fovv 100x100, slice thickness 3mm.

Axial: T1- weigthed Spin Echo (T1 SE) TR 435 TE 13; matrix 320x320: aquisition time 3:10; FOV 100x100; slice thickness 3mm.

Sagittal: fat saturated protone weighted turbo spin echo (PD FS) TR 3500 TE 38; matrix 320x320; acquisition time 3:20; FOV 100x100, slice thickness 3mm.

Sagittal: T2-weighted Turbo Spin Echo TR 7280 TE106; matrix 320x320; acquisition time 4:45; FOV 100x100; slice thickness 3mm.

In this retrospective analysis, the MRI examinations were evaluated on a digital workstation [IMPACS EE, Agfa, Bonn Germany] with two high resolution monitors [Barco GmbH, Karlsruhe, Germany].

Surgery was performed by two experienced senior surgeons (MJR, SW) with a personal experience of more than five years of shoulder arthroscopy. MJR performed 46 (67%) and SW 23 (33%) of all cases. The glenohumeral joint was inspected by introducing the 30° angled scope in a posterior standard portal. The forearm was positioned and moved on the trimano® (Arthrex) arm holder. The shoulder was assessed in a standardised fashion to evaluate the labrum, LHB, pulley system and the entire rotator cuff, especially the subscapularis tendon. The arm was moved during this step to evaluate biceps stability and to avoid missing non-dislocated tears. The subscapularis tendon was assessed with the 30° scope by bringing the arm in anteversion and internal rotation to screen for hidden, instable tears or grade 2 or 3 tears. Then, the subacromial space was inspected. A lateral portal was established for introducing the shaver and bursectomy was performed. Afterwards the scope was switched from the posterior to the lateral portal to evaluate the rotator cuff by a probe with a clear view on the rotator cuff. All patient data was pseudonymised. Local institutional ethical review board (IRB) approval was obtained (“ethics commission of the university hospital Regensburg”; nr. 19-1452-101).

We established an Excel® [Microsoft, Redmond, USA] sheet for assessing shoulder MRI in a standardised fashion. The subscapularis was evaluated according to the classification by Fox/Romeo^10^. For the rotator cuff we asked to describe the tear extent as either partial tear according to the Ellman’s classification^11^ or full thickness tear according to Snyder *et al*^12^. The location and extent of the tear had to be specified for partial tears with A (articular sided) and B (bursal sided). Type C transmural tears had to be located (supraspinatus or infraspinatus) and described with C 1-3. C 4 type tears were massive ruptures involving more than two tendons according to Snyder^12^. Medial tendon retraction was assessed by the Patte classification^13^. The labrum was screened for 4 pathologies or findings (bony Bankart lesion, cartilage Bankart lesion, labral tear/dislocation or sublabral foramen). The biceps tendon was to be screened for a possible SLAP- lesion (Yes or No only, without further classification) and a pulley- lesion or instability according to Habermeyer^14^.

This standardised assessment of the MRI was explained to each study rater prior to study start. Rater 1 (BH) is a sports orthopaedic surgeon (>13 years of clinical experience). Rater 2 (PH) is a diagnostic musculo-skeletal radiologist (>16 years of clinical experience). Rater 3 (MJR) is a sports orthopaedic surgeon (>13 years of clinical experience).

The MRI images were unnamed (pseudonymised) and stored on CD prior to study start and enumerated 1-69. The CDs were given to rater 1 and rater 2 in a blind assignment to evaluate the MRIs twice, at t1 and t2 (=t1 + 6 weeks). Rater 3 gathered all findings from shoulder surgery of each study participant by reviewing arthroscopy images, letters and surgery reports, which served as positive control. All findings were documented by each rater in separate EXCEL® [Microsoft, Redmond, USA] work sheets.

Data of the two raters ‘assessments at two timepoints were compared to positive control. Data were the involvement of substructures and the extent of injuries. The raters followed a two-stage assessment. First, is there a pathology (yes=1, no=0)? Second, the accuracy of classifying the pathology was detected (categorical data) on a numerical scale (0,1,2,3 etc.). The mean difference (=disagreement) between raters and positive control was given as score. A low score means a low disagreement (=high accuracy). Continuous data were summarised as means +/- standard deviations or as medians [25th and 75th percentiles] as appropriate.

The intra-observer disagreement was used to assess a difference between timepoints. It is calculated within each rater’s assessments. The differences were averaged across raters. The inter-observer difference is used to examine the disagreement between the positive control, rater 1 and rater 2. Differences between the measurements of all instances were averaged.

The methods are described in the script Biostatistics for Biomedical Research by Harrell and Slaughter^15^. Inter- and intra-observer disagreement estimates, and 95% confidence intervals are based on bootstrap calculations. The summary statistics are shown in Tables I-IV stratified by rater and time of assessment. Disagreement rates are given, and bootstrap confidence intervals are shown in brackets. All calculations were performed with the statistical analysis software R (R Core Team, 2020).

**Table I:** Rotator cuff.

	N N=69	PC N=69	R1/T1 N=69	R1/T2 N=69	R2/T1 N=69	R2/T2
Subscapularis	343	0.12 (8)	0.28 (19)	0.29 (20)	0.42 (29)	0.41 (28)
Disagreement to positive control			0.257 (0.162, 0.353)		0.341 (0.232, 0.449)	
Inter-observer disagreement				0.301 (0.206, 0.397)		
Intra-observer disagreement				0.087 (0.043, 0.138)		
Subscapularis (Fox/Romeo): 0	345	0.88 (61)	0.72 (50)	0.71 (49)	0.58 (40)	0.59 (41)
1		0.09 (6)	0.14 (10)	0.16 (11)	0.20 (14)	0.17 (12)
2		0.01 (1)	0.10 (7)	0.09 (6)	0.10 (7)	0.12 (8)
3		0.01 (1)	0.01 (1)	0.01 (1)	0.06 (4)	0.06 (4)
4		0.00 (0)	0.01 (1)	0.03 (2)	0.06 (4)	0.06 (4)
Disagreement to positive control			0.442 (0.297, 0.594)		0.717 (0.486, 0.986)	
Inter-observer disagreement				0.645 (0.442, 0.891)		
Intra-observer disagreement				0.21 (0.13, 0.304)		
Supraspinatus: yes	343	0.61 (42)	0.62 (42)	0.71 (48)	0.65 (45)	0.68 (47)
Disagreement to positive control			0.294 (0.199, 0.397)		0.261 (0.159, 0.37)	
Inter-observer disagreement				0.228 (0.14, 0.324)		
Intra-observer disagreement				0.072 (0.029, 0.123)		
Infraspinatus: yes	345	0.06 (4)	0.06 (4)	0.01 (1)	0.13 (9)	0.12 (8)
Disagreement to positive control			0.051 (0.014, 0.101)		0.181 (0.094, 0.275)	
Inter-observer disagreement				0.145 (0.072, 0.225)		
Intra-observer disagreement				0.029 (0.007, 0.058)		
Articular sided: yes	345	0.12 (8)	0.12 (8)	0.07 (5)	0.12 (8)	0.13 (9)
Disagreement to positive control			0.167 (0.094, 0.246)		0.181 (0.101, 0.275)	
Inter-observer disagreement				0.123 (0.065, 0.188)		
Intra-observer disagreement				0.072 (0.029, 0.123)		
Bursal sided: yes	345	0.16 (11)	0.19 (13)	0.28 (19)	0.19 (13)	0.20 (14)
Disagreement to positive control			0.275 (0.174, 0.377)		0.167 (0.087, 0.261)	
Inter-observer disagreement				0.268 (0.174, 0.37)		
Intra-observer disagreement				0.051 (0.022, 0.087)		
Transmural: yes	345	0.35 (24)	0.30 (21)	0.35 (24)	0.33 (23)	0.36 (25)
Disagreement to positive control			0.152 (0.072, 0.239)		0.116 (0.051, 0.196)	
Inter-observer disagreement				0.167 (0.087, 0.254)		
Intra-observer disagreement				0.036 (0.007, 0.072)		
Extent (1-3): 0	343	0.37 (25)	0.39 (27)	0.30 (21)	0.48 (33)	0.48 (33)
1		0.22 (15)	0.16 (11)	0.04 (3)	0.39 (27)	0.38 (26)
2		0.09 (6)	0.28 (19)	0.28 (19)	0.06 (4)	0.07 (5)
3		0.30 (20)	0.17 (12)	0.38 (26)	0.07 (5)	0.07 (5)
4		0.01 (1)	0.00 (0)	0.00 (0)	0.00 (0)	0.00 (0)
Disagreement to positive control			0.813 (0.627, 1.007)		0.813 (0.627, 1.015)	
Inter-observer disagreement				0.978 (0.797, 1.159)		
Intra-observer disagreement				0.268 (0.181, 0.362)		
Retraction (Patte): 0	345	0.72 (50)	0.68 (47)	0.72 (50)	0.75 (52)	0.75 (52)
1		0.14 (10)	0.16 (11)	0.12 (8)	0.12 (8)	0.10 (7)
2		0.10 (7)	0.10 (7)	0.10 (7)	0.07 (5)	0.09 (6)
3		0.03 (2)	0.06 (4)	0.06 (4)	0.06 (4)	0.06 (4)
Disagreement to positive control			0.239 (0.138, 0.355)		0.297 (0.181, 0.428)	
Inter-observer disagreement				0.319 (0.196, 0.457)		
Intra-observer disagreement				0.058 (0.022, 0.101)		

N is the number of non-missing values. Numbers after proportions are frequencies. Disagreement between rater (positive control) and within the rater. Numbers in brackets for disagreement rates are bootstrap confidence intervals. PC=positive control, R= rater, T= time

**Table II:** Labral pathologies.

	N	PC N=69	R1/T1 N=69	R1/T2 N=69	R2/T1 N=69	R2/T2 N=69
Labrum: yes	345	0.09 (6)	0.13 (9)	0.10 (7)	0.17 (12)	0.17 (12)
Bankart lesion (labrum): yes	343	0.09 (6)	0.01 (1)	0.03 (2)	0.09 (6)	0.09 (6)
Disagreement to positive control			0.08 (0.022, 0.145)		0.088 (0.029, 0.162)	
Inter-observer disagreement				0.081 (0.022, 0.154)		
Intra-observer disagreement				0.007 (0, 0.022)		
Bankart lesion (bony): yes	343	0.00 (0)	0.01 (1)	0.01 (1)	0.00 (0)	0.00 (0)
Disagreement to positive control			0.014 (0, 0.043)		0 (0, 0)	
Inter-observer disagreement				0.015 (0, 0.044)		
Intra-observer disagreement				0 (0, 0)		
Sublabral foramen: yes	340	0.00 (0)	0.10 (7)	0.07 (5)	0.09 (6)	0.09 (6)
Disagreement to positive control			0.087 (0.036, 0.152)		0.09 (0.03, 0.164)	
Inter-observer disagreement				0.179 (0.097, 0.269)		
Intra-observer disagreement				0.029 (0.007, 0.058)		

N is the number of non-missing values. Numbers after proportions are frequencies. Disagreement between rater (positive control) and within the rater. Numbers in brackets for disagreement rates are bootstrap confidence intervals. (PC: positive control, R: rater, T: time)

**Table III:** Biceps pathologies.

	N	PC N=69	R1/T1 N=69	R1/T2 N=69	R2/T1 N=69	R2/T2 N=69
Biceps: yes	345	0.26 (18)	0.39 (27)	0.41 (28)	0.51 (35)	0.51 (35)
Disagreement to positive control			0.341 (0.246, 0.435)		0.478 (0.362, 0.594)	
Interobserver disagreement				0.413 (0.312, 0.514)		
Intra-observer disagreement				0.123 (0.072, 0.174)		
SLAP-lesion: yes	339	0.01 (1)	0.04 (3)	0.03 (2)	0.16 (11)	0.18 (12)
Disagreement to positive control			0.037 (0.007, 0.075)		0.159 (0.076, 0.25)	
Interobserver disagreement				0.191 (0.11, 0.279)		
Intra-observer disagreement				0.043 (0.014, 0.08)		
Pulley-lesion: yes	323	0.25 (17)	0.44 (26)	0.40 (27)	0.43 (28)	0.44 (28)
Disagreement to positive control			0.326 (0.227, 0.424)		0.422 (0.312, 0.547)	
Inter-observer disagreement				0.389 (0.286, 0.492)		
Intra-observer disagreement				0.109 (0.065, 0.159)		
Type (Habermeyer‘s classification) : 0	324	0.74 (51)	0.56 (33)	0.60 (40)	0.57 (37)	0.56 (36)
1		0.12 (8)	0.08 (5)	0.04 (3)	0.12 (8)	0.12 (8)
2		0.06 (4)	0.10 (6)	0.15 (10)	0.15 (10)	0.16 (10)
3		0.06 (4)	0.22 (13)	0.16 (11)	0.06 (4)	0.06 (4)
4		0.03 (2)	0.03 (2)	0.04 (3)	0.09 (6)	0.09 (6)
Disagreement to positive control			0.866 (0.642, 1.104)		1.031 (0.754, 1.338)	
Inter-observer disagreement				1.095 (0.833, 1.357)		
Intra-observer disagreement				0.319 (0.188, 0.457)		

N is the number of non-missing values. Numbers after proportions are frequencies. Disagreement between rater (positive control) and within the rater. Numbers in brackets for disagreement rates are bootstrap confidence intervals. (PC: positive control, R: rater, T: time).

**Table IV:** Inter-rater comparison of diagnostic accuracy (differences between raters’ disagreement to positive control).

Variable	P
Subscapularis tear	0.037*
Fox/Romeo classification	0.003*
Supraspinatus tear	0.393
Infraspinatus tear	< 0.001*
Articular sided partial tears	0.549
Bursal sided partial tears	0.005*
Transmural tear	0.164
Extent of transmural tear	0.805
Retraktion of transmural tear (Patte classification)	0.088
Labral leasion	0.409
Bony Bankart lesion	> 0.999
Sublabral foramen	0.986
Biceps pathology	0.005*
SLAP lesion	< 0.001*
Pulley lesion of biceps tendon	0.052
Classifying pulley lesion (Habermeyer)	0.022*

Variables= study variables; P: differences of diagnostic accuracy between raters in comparison to positive control (p<0.05 is significant) *: statistical significant with p<0.05

Statistical significance level was alpha = 0.05. Differences between raters’ disagreement to positive control were evaluated using mixed logistic and mixed ordinal regression models. Table IV shows p-values to demonstrate significant differences for each study variable between rater 1 and 2.

## Results

Twenty-five women (36%) and forty-four men (64%), aged between 26 and 83 years, were included into the study. The mean age at surgery was 53.3 years (SD ±11.4). We observed 54 rotator cuff tears (78%) among study population (n=69) according to positive control. The distribution of tears was as follows: supraspinatus (SSP, n=42), subscapularis (SSC, n=8) and infraspinatus (ISP, n=4). SSP and ISP (n=46) were further rated by positive control with 24 transmural tears and 19 partial tears (8 articular sided tears, 11 bursal sided). Full thickness tears were mostly smaller type C1 (n=15) or larger C3 (n=20) (see Table I for details).

Both raters detected tear location with supraspinatus (71% vs 74%) and infraspinatus tears (94% vs 81%) with good accuracy (rates of agreement) compared to positive control. The inter-observer disagreement was 22.8% for supraspinatus and 14.5% for infraspinatus with a low intra-observer variability at both timepoints (T1 and T2).

The specification of tear extent was solid by both raters for partial tears. Articular sided tears were assessed correctly in >80% by both raters. Bursal sided tears were detected with 72% accuracy by rater 1 and 83% by rater 2, respectively. Furthermore, transmural tears were evaluated highly accurately with 84% for rater 1 and 88% for rater 2. All these assessments had a very low intra-observer variability.

However, there were difficulties in assessing transmural tear extent in more detail with very high disagreement to positive control and also high inter-observer disagreement. Also, the small number (n=4) of tears involving the ISP allows only limited interpretation of the data. The medial tendon retraction was evaluated more consistently and accurately, compared to intra-operative findings, by both raters. Still, there was an elevated inter-observer disagreement (0.319). For further details see Table I.

There were deviating results for detection of subscapularis tendon tears. On the one hand, there were only 8 patients with tears, which were described during surgery, according to rater 3, which served as positive control (n=6 with Fox/Romeo type 1, n=1 of type 2 and n=1 of type 3 tears). In comparison, the two raters detected a higher and also consistent number of tears by evaluating the MRI images at both time points (t1 and t2). Rater 1, the orthopaedic surgeon, identified 19 (t 1) and 20 (t2) SSC tears, whereas rater 2, the radiologist, identified 29 (t1) and 28 (t2) tears on the MRI images. The rates of agreement to positive control were therefore moderately high with 74% for the orthopaedic surgeon (rater 1) and only 65% for the radiologist (rater 2). Grading of the tears, by using the Fox/Romeo classification, was also inconsistent by both raters compared to positive control with elevated disagreement rates (0.442 vs 0.717). Due to the small number of eight confirmed SSC tears the data should be carefully interpretated.

Labral findings were detected in only 6 of 69 study patients (9%) according to surgery reports. Both raters were able to identify labral pathologies with good accuracy (92% vs 91%) and a low intra-observer variability (<1%) on MRI images. Rater 1 falsely identified one bony Bankart’s lesion. Both raters misinterpreted a sublabral foramen for a labral lesion. Intra-operatively only one SLAP lesion was identified. The MRI assessment had a high accordance rate for this one detected SLAP lesion (96.3% for rater 1, 84.1% for rater 2). Results and agreement rates should be interpretated with caution due to the rather small sample size for labrum and SLAP. For further details see Table II.

Seventeen pulley-lesions of the LHB with instability of 69 (25%) study patients were found during surgery. The correct pre-operative assessment of the pulley lesions on MRI images was weak by both raters (66.4% for rater 1 vs 57.2% for rater 2). There was a high inter-observer (38.9%) and intra-observer (10.9%) variability for pulley lesions. Grading of pulley lesions, according to Habermeyer’s classification, was very inconsistent to positive control for both raters. The intra-observer variability was also very high. For further details see Table III.

Both raters demonstrated good agreement rates, compared to positive control, in numerous anatomical structures. Due to the small sample size and low numbers of certain pathologies there were only indications of different diagnostic accuracy, but no clear differences. The agreement rates for subscapularis and infraspinatus tears was mildly higher for rater 1. Bursal sided partial tears of the rotator cuff were assessed slightly more precisely by the radiologist. Both raters deviated in results to positive control in assessment of subscapularis tears and classifying of biceps pulley lesions. For further details see Table IV.

## Discussion

Although ultrasound has a high accuracy in detecting rotator cuff and labral pathologies^16,17^, MRI and MRA (MR arthrography) are often used prior to surgery, as they have a comparable high diagnostic value for evaluating shoulder injuries^18,19^.

The aim of this retrospective study was to examine how reliable shoulder MRI images can be assessed as this has direct consequences for the patient and surgeon alike. We intended to use a setup that is very close to daily routine of a specialised department. The data and findings during shoulder surgery and post-surgery treatment offer valuable feedback, which can be applied as positive control to evaluate diagnostic accuracy and possibly improve treatment planning. Furthermore, we were interested whether there was a different diagnostic assessment between an orthopaedic surgeon and a radiologist. We aimed to investigate and interpret varying assessments carefully by analysing agreements of the observers, rather than calculating absolute rates of right and wrong answers.

Our study demonstrated an accuracy of detecting transmural rotator cuff tears of 84% and 89% and partial tears between 70-80% for both raters. Although these numbers are slightly lower than results in the literature^20,21^, it confirms that partial tears are assessed less precisely (with lower sensitivity) than full thickness tears. Smith *et al* examined 44 studies in a meta-analysis and detected a pooled MRI sensitivity for partial cuff tears of 80% and for transmural tears 91%^21^. A 2019 meta-analysis found MRA and MRI equally effective for assessing bursal sided partial rotator cuff tears^22^. New advanced MRI techniques were examined by Lazik-Palm *et al*^23^ with both, a pre-operative 7 Tesla shoulder MRI and arthroscopic surgery. They established, in a small number of patients, a sensitivity of 86% (specificity 74%) for all structures, which is lower than 3 T MRI results^19,22^ and also described a bias of overrating signal alterations of the tendon and to mind the magic angle effect^23^.

The assessment of subscapularis tears in pre-operative MRIs is challenging. We only had a very small number (n=8) of intra-operatively confirmed SSC tears in our study sample. Rater 1 and 2 detected a higher number of tears (ca. 20 vs 28) on the MRI images. This could either mean that tears were overinterpreted by rater 1 and 2, or that a certain number of SSC tears were missed by the surgeons. We used a 30°, and not a 70° scope. As a direct consequence of these findings, we slightly alternated our diagnostic assessment during arthroscopy by using the 30° scope and bringing the arm in anteversion and internal rotation, and optionally with ventral pressure on the humeral head, to evaluate SSC in more detail. The arm is rotated under visual control of the subscapularis and the insertion area is probed from proximal to distal on both sides of the tendon. The follow-up reports of study patients to not indicate enduring subscapularis pain, although this study was not specifically designed for a standardised follow-up. A recent systemic review for diagnosing subscapularis tears established a sensitivity of only 0.68 (95%CI 0.64-0.72) for MRI^24^. This limited diagnostic precision for subscapularis tears might be an explanation why both raters of our study had difficulties of correctly classifying detected tears. Furthermore, we intended to use the findings during surgery as feedback for our study. The Fox/Romeo classification for SSC tears is a rather surgical classification and potentially only limited suitable for describing tears on MRI images.

Labral pathologies are common and can even be detected in 25% of shoulders of asymptomatic professional and collegiate ice hockey players^1^. A 2019 systematic review and meta-analysis found MRI to be the best diagnostic modality for acute labral pathologies with a sensitivity of 0.77 (95% CI 0.70-0.84)^25^. Despite the small number (n=6 of 69) of cases in our study, both raters established a good accuracy of more than 90% correctly identified labral injuries and >80% agreement for a one SLAP lesion, although we used a non-contrast MRI.

Pathologies of the LHB (17 pulley lesions) were detected with low rates of agreement (66.4% vs 57.2%) on 1.5 T MRI images by our raters with a high inter-rater disagreement. Further classifying the pulley-lesion was very inconsistent. These results confirm Baptista *et al*^26^ who had a comparable study design and also found only a moderate overall accuracy of 1.5 T MRI for detecting LHB tears (71%-73%) and LHB tendon displacement (51%-58%). MR-arthrography seems to be controversial as Loock *et al*^27^, found only a low accuracy of MRA for LHB pathologies and would not recommend it for pre-surgery imaging. Contrary, Barile *et al* recommend a 1.5 T MRA with the arm in the ABER position for pulley lesions^28^. Kim *et al*^29^ described a high diagnostic accuracy of a pre-operative 3 Tesla MRI for LHB pathologies.

The inter-observer comparison, orthopaedic surgeon vs radiologist, detected only small significant differences of diagnostic accuracy and agreement rates. Subscapularis and infraspinatus tendon tears were detected slightly more accurate by the orthopaedic surgeon, although the sample size was small. Bursal sided tears were assessed more accurately by the radiologist. A further interpretation of these findings seems to be speculative due to the small study population.

A recent study established that information on the patient’s clinical complaints is helpful of better interpreting shoulder MRI as degenerative findings seem to be age-related and very common^30^. Yazigi *et al* demonstrated that experience of orthopaedic shoulder surgeons and musculoskeletal radiologists ensures higher results in MRI diagnostics^31^.

A meta-analysis of 44 studies by Smith *et al* described no difference of diagnostic accuracy between a musculoskeletal radiologist and a general radiologist in shoulder MRI for rotator cuff injuries^21^. This current study has limitations. First, we only had a small study population (n=69) with few cases of certain pathologies (i.e. one SLAP lesion). A larger cohort is necessary to further explore and confirm our current findings. Second, the intra-operative assessment could have been inaccurate and of low quality as positive control. To avoid that, all surgeons followed standardised diagnostic steps and are experienced in their field. One of the two surgeons (MJR) of this study served as rater 3 (=positive control). Rater 1 (BH), an orthopaedic surgeon, was present during some of the surgeries. We conducted our study in a pseudonymised fashion and kept a timely gap between surgeries and our study (>5 months) to limit a possible bias. The results did not indicate that rater 1 had advantage over rater 2.

Third, we initiated this analysis to learn from our daily routine. This meant that we assessed the diagnostic accuracy of the raters inversely by learning from the surgery images, reports and patient files and using this as control. Radiologic classifications often cannot be fully transferred to surgical findings, which leads to a certain indistinctness.

The idea for this study was to re-evaluate if surgery was indicated correctly and if findings of MRI images were either confirmed, completely missed or graded differently during surgery. From a surgeon’s point of view, the interpretation of MRI images should conclude practical implications: Does the pathology or extent of finding require surgery (i.e. subscapularis tear)? If so, is tendon reconstruction still possible, how much surgery time or numbers of implants should be planned etc.? Do other structures need to be addressed? How should the patient be educated?

Forth, this study used 1.5 Tesla non-contrast MRI images. This represents a realistic daily routine in our clinic as many patients already had MRI prior to consulting a shoulder specialist. A high field MRI or MRA might increase diagnostic accuracy for certain pathologies^21,28^. A recent study suggests that saline could be used instead of gaudolinum for MRA with equal results in detecting labral and rotator cuff pathologies^32^. In the near future, software algorithms could support orthopaedic surgeons and radiologist by analysing all diagnostic modalities to further enhance diagnostic accuracy^33^.

## Conclusion

In summary, this study aimed to learn from possible different assessments of MRI images by a radiologist or orthopaedic surgeon to improve our daily routine, rather than drawing any conclusions by direct comparison. We found that tears of the rotator cuff and labral pathologies can be assessed in shoulder MRI images with a high accuracy compared to intra-operative findings. A 1.5 Tesla MRI, with supine position of the arm, offered only moderate sensitivity for detecting pulley lesions of the LHB. There were only small differences between a radiologic and orthopaedic interpretation of the images. Future studies with larger study groups can further explore these results.
